# Indication of metabolic inflexibility to food intake in spontaneously overweight Labrador Retriever dogs

**DOI:** 10.1186/s12917-019-1845-5

**Published:** 2019-03-20

**Authors:** Josefin Söder, Sara Wernersson, Johan Dicksved, Ragnvi Hagman, Johnny R. Östman, Ali A. Moazzami, Katja Höglund

**Affiliations:** 1 Department of Anatomy, Physiology and Biochemistry, Faculty of Veterinary Medicine and Animal Science, Swedish University of Agricultural Sciences, Box 7011, 75007 Uppsala, Sweden; 2 Department of Animal Nutrition and Management, Faculty of Veterinary Medicine and Animal Science, Swedish University of Agricultural Sciences, Box 7024, 75007 Uppsala, Sweden; 3 Department of Clinical Sciences, Faculty of Veterinary Medicine and Animal Science, Swedish University of Agricultural Sciences, Box 7054, 75007 Uppsala, Sweden; 4 Department of Molecular Sciences, Faculty of Natural Resources and Agricultural Sciences, Swedish University of Agricultural Sciences, Box 7015, 75007 Uppsala, Sweden

**Keywords:** Acetylcarnitine, Canine, Carnitine, Fatty-acid oxidation, Feed-challenge test, LC-TOFMS, Lipidomics, Liquid chromatography-time of flight mass spectrometry, Obesity, Phospholipids

## Abstract

**Background:**

Obesity in dogs is an increasing problem associated with morbidity, shortened life span and poor life quality. Overweight dogs exhibit postprandial hyperlipidaemia, highlighting the need to identify potential dysregulations in lipid metabolism. This study investigated metabolites related to lipid metabolism (i.e. acylcarnitines and taurine) and phospholipids in a feed-challenge test and aimed to identify metabolic variations in spontaneously overweight dogs. Twenty-eight healthy male Labrador Retriever dogs were included, 12 of which were classified as lean (body condition score (BCS) 4–5 on a 9-point scale) and 16 as overweight (BCS 6–8). After overnight fasting (14–17 h), fasting blood samples were collected and dogs were fed a high-fat meal followed by postprandial blood sample collection hourly for 4 h. Liquid chromatography-time of flight mass spectrometry (LC-TOFMS) was used to identify plasma metabolites and phospholipids. Multivariate models, mixed model repeated measures and linear regression analyses were used for data interpretation.

**Results:**

In all dogs, propionylcarnitine, stearoylcarnitine and nine phospholipids increased in response to food intake, while vaccenylcarnitine decreased (*P* ≤ 0.005 for all). Overall, carnitine and acetylcarnitine signal areas in the feed-challenge test were lower in overweight dogs (*P* ≤ 0.004). Notably, fasting plasma acetylcarnitine was lower in overweight dogs than in lean dogs (*P* = 0.001) and it did not change in response to feeding. The latter finding was in contrast to the decreased acetylcarnitine signal area found in lean dogs at one hour postprandially (*P* < 0.0001). One fasting phosphatidylcholine (PCaa C38:4) was higher in prominently overweight dogs (BCS > 6) than in lean dogs (*P* < 0.05).

**Conclusions:**

Plasma carnitine status was overall lower in spontaneously overweight dogs than in lean dogs in this cohort of healthy Labrador Retriever dogs, indicating a potential carnitine insufficiency in the overweight group. The acetylcarnitine response in overweight dogs indicated decreased fatty acid oxidation at fasting and metabolic inflexibility to food intake. Further studies on metabolic inflexibility and its potential role in the metabolism of overweight dogs are warranted.

**Electronic supplementary material:**

The online version of this article (10.1186/s12917-019-1845-5) contains supplementary material, which is available to authorized users.

## Background

There has been a rapid increase in the number of overweight and obese humans world-wide. Obese humans often suffer from severe health complications and obesity is nowadays classified as a disease [1]. Dogs largely share lifestyle with their owners [2, 3] and overweight is also an increasing problem in dogs [4, 5], associated with morbidity, poor life quality [6, 7] and shorter life expectancy [8, 9]. Overweight dogs have been shown to display insulin resistance and postprandial hypertriglyceridaemia [10–12] and variations in metabolites related to lipid metabolism [13–15] compared with lean dogs.

In humans, it has been proposed that metabolic inflexibility could be a link between obesity and insulin resistance [16, 17]. Such a link might also be present in overweight insulin-resistant dogs. The importance of a flexible metabolism has gained growing attention in metabolic health and obesity research, as it describes mitochondrial oxidation rate and fuel switch capacity [18–20]. Carnitine is considered an important metabolite in the regulation of metabolic flexibility and in lipid metabolism as it mediates the import of long- and medium-chain fatty acids into the mitochondrial matrix for beta-oxidation and acts as a buffer for acyl groups by formation of carnitine esters, i.e. acylcarnitines [17, 21]. In humans, acylcarnitine patterns analysed by mass spectrometry are commonly used for screening and diagnosis of congenital defects in lipid metabolism [22]. Moreover, carnitine has been shown to play a role in complicated type 2 diabetes mellitus in humans [23] and elevated medium- or long-chain acylcarnitine concentrations have been found in obese compared with lean subjects [24, 25]. Acylcarnitine patterns after food intake have been investigated in humans [26], but corresponding studies in dogs are currently lacking.

In overweight humans, lipoprotein fractions have been shown to be higher than in lean subjects, except for high-density lipoproteins which are generally lower in obesity [27]. Whether all or specific lipoprotein fractions increase in overweight dogs is unclear, as previous studies show diverging results [28–31]. In addition, dogs have an extra high-density lipoprotein fraction compared with humans [28]. Phospholipids constitute the main component of lipoproteins in dogs, [32] and are therefore of special interest. Lipidomics is an analytical approach that can be used to characterise the pattern of phospholipids in plasma [33], but in dogs this approach has so far mainly been used in acutely overweight subjects [14]. Altered lipid pathways in obese humans, such as increases in lysophosphatidylcholines, have been identified with lipidomics [34]. It is therefore of interest to compare phospholipid profiles between lean and overweight dogs using liquid chromatography-time of flight mass spectrometry (LC-TOFMS) lipidomics.

Our starting hypothesis was that before and after food intake, acylcarnitine metabolites and potentially certain phospholipids are altered similarly in overweight dogs as in overweight humans. The aims of the present study were therefore to investigate metabolites related to lipid metabolism (i.e. acylcarnitines and taurine), and phospholipids in a feed-challenge test and to identify metabolic variations related to spontaneous overweight in dogs.

## Methods

### Dogs and general study design

Privately-owned intact male Labrador Retriever dogs were recruited by personal letters to dog owners using a register provided by The Swedish Kennel Club. The selection process consisted of an on-line survey and a clinical health examination (performed by JS) including blood and urine analyses, all to ensure that the dogs met the inclusion and exclusion criteria [35]. Twenty-eight healthy Labrador Retriever dogs were included in the study. In addition to the health examination, all dogs underwent body condition scoring (BCS), were weighed and photographed [35].

No adjustments were made to the dogs’ regular home diet or treats prior to participation. During two weeks preceding the study, dietary history was acquired by daily food diaries. According to those, all dogs had their main energy supply from dry or wet complete commercial diets and the most common protein source was chicken. A limited number of lean and overweight dogs were fed a low-fat calorie-restricted diet, obtained their main part of metabolisable energy from fat or were given complete diets containing L-Carnitine additives. The frequencies, with which lean and overweight dogs were awarded table scraps, treats or dog chews did not differ (Additional file 1).

Dogs were fasted from 6 pm on the day before clinical samplings and in the morning of the examination day water was withheld and a voided urine sample was taken. Between 8 and 9:30 am, after 14–17 h of fasting, the dogs arrived at the clinic, underwent the health examination and fasting blood samples were taken. Thereafter a test meal was served and postprandial blood samples were collected hourly four times. The study was performed at the Swedish University of Agricultural Sciences, Uppsala, Sweden and was approved by the Ethics Committee for Animal Experiments, Uppsala, Sweden (C180/12). This prospective study followed guidelines for reporting observational studies in epidemiology [36] and written consent of the owner was obtained for each dog.

### Assessment of body condition

The dogs were assigned a BCS by the same veterinarian (JS) according to a 9-point scale and the cut-off for overweight (BCS ≥ 6) as suggested by the scoring scale was applied [37]. Based on BCS, the lean group (BCS 4–5) consisted of 12 dogs and the overweight group (BCS 6–8) consisted of 16 dogs. Body weight differed significantly between body condition groups (*P* = 0.004), while age and ideal body weight did not [35]. Serum leptin concentrations were higher in overweight than in lean dogs [35] and the assigned BCS was positively associated with fasting leptin concentration (linear regression R^2^ = 0.41, *P* < 0.0001) (Additional file 2).

### Feed-challenge test and blood sample collections

In the feed-challenge test, all dogs were given half their daily energy requirement (DER) as a high-fat mixed-meal. The equation used to compute DER (131 kcal × body weight _kg_^0.75^) was a prediction equation from existing data on adult Labrador Retriever dogs [38]. In the DER equation, the actual body weight of lean dogs and the calculated ideal body weight [10, 37] of overweight dogs was used. The amount of test feed in grams given to lean and overweight dogs did not differ [35]. The test meal was weighed and served with water added (same amount in grams as the individual test meals). The test feed (Science PlanTM Canine Adult Performance, Hills, Etten Leur, Netherlands) provided 4230 kcal/kg, with 51% of the metabolisable energy (ME) as fat, 26% as carbohydrate and 23% as protein. Taurine, omega-3 and omega-6 fatty acids, betacarotene and vitamin A, C, D and E were added (composition of macronutrients and other dietary components presented as-fed, by the manufacturer). Macronutrient composition and calculated ME (as-fed) of the batch of test feed were confirmed by an independent authorised laboratory (Additional file 3). All 28 dogs voluntarily consumed all food and water within 10 min of being served and the postprandial period started at the first bite. The dogs were given nothing further to eat or drink until completion of the postprandial samplings.

Fasting blood samples were collected by a venous catheter 15 min before the test meal and then hourly for up to four hours postprandially. Details on the blood sample procedure and serum collection have been previously described [39]. Plasma tubes (Hettich Vacuette Lithium Heparin, Greiner bio-one) were centrifuged (1500×*g* for 10 min) directly after sampling and plasma was transferred to polypropylene tubes (SC Micro Tube PCR-PT, Sarstedt AG & Co, Nümbrecht, Germany) and immediately frozen at − 70 °C.

### LC-TOFMS-based metabolomics analysis

#### Sample preparation

For LC-TOFMS, the plasma samples were thawed on ice and 5 μL were extracted in 495 μL of methanol (LC-MS grade, VWR Chemicals, Radnor, PA) spiked with phospholipid standards (Additional file 4). The protein precipitate was separated by centrifugation at 10000×*g* for 10 min at 4 °C and 450 μL supernatant were transferred to chromatography vials.

#### LC-TOFMS analysis

The chromatography was performed on an Agilent 1100 high-performance liquid chromatograph (HPLC) (Agilent Technologies, Santa Clara, CA). The aquatic mobile phase A consisted of 10 mM ammonium formate (Fluka analytical, Sigma-Aldrich, St. Louis, MO) with 0.1% formic acid (vol:vol) (Sigma-Aldrich). Mobile phase B consisted of acetonitrile (Gradient grade, Merck, Darmstadt, Germany) with 0.1% formic acid (vol:vol). The following gradient, expressed as the relative fraction of mobile phase B, was used: 0 min 95%, 0.5 min 95%, 10.5 min 40%, 15 min 40%, 17 min 95% and 32 min 95%. A 10 μL aliquot of extract was injected onto a Waters Atlantis hydrophilic interaction liquid chromatography (HILIC) Silica column (3 μm, 2.1 × 150 mm) equipped with a 10 mm guard column of the same composition. The flow rate was 0.25 mL/min and the column oven was kept at 30 °C. The time-of-flight mass spectrometer (Bruker maXis impact, Bruker Daltonics, Bremen, Germany) was operated in positive ionisation mode with a plate offset voltage of 500 V and a capillary voltage of 4 kV. Nitrogen gas heated to 200 °C was administered at 8 L/min with a nebuliser pressure of 2 bar for desolvation. The digitiser sample rate was set at 4 GHz and profile sample spectra were collected at a rate of 1 Hz in a constrained randomisation run in order to minimise drift bias [40].

#### Data processing

The data were processed using Compass DataAnalysis 4.1 (Bruker Daltonics, Bremen, Germany) and the chromatograms were smoothed by one cycle of Gaussian smoothing with a width of 2 s prior to area calculation. The chromatographic peak of each metabolite was detected using its accurate mass and retention time including a mass window of ±0.01 *m/z* and a retention time window of ±30 s. For carnitines, relative intensities were determined. For phospholipids, the absolute concentrations in plasma were determined against four different phospholipid internal standards added to the samples.

### Statistical analyses

#### Metabolites

**Fig. 1:**
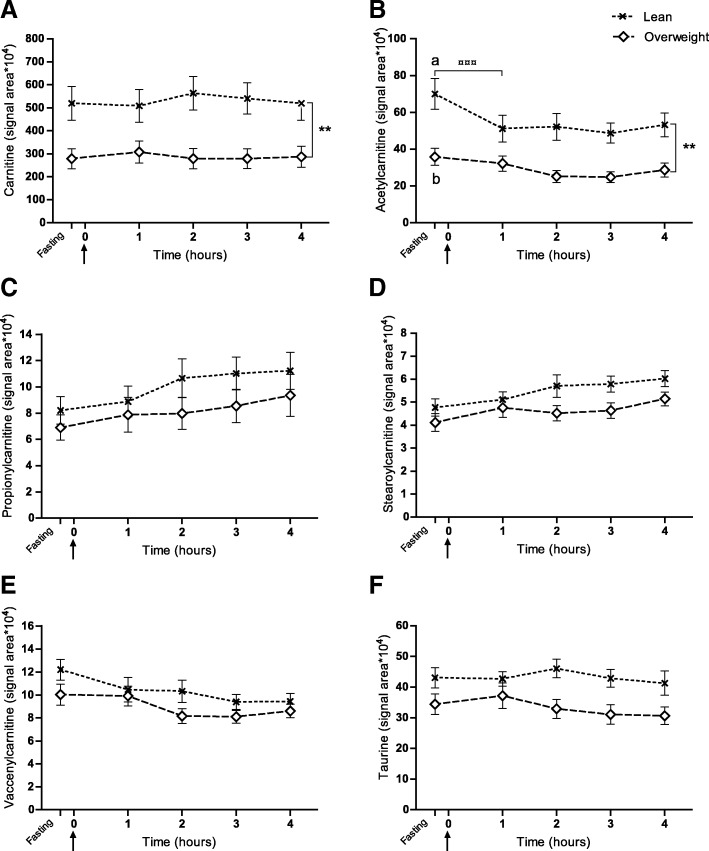
Metabolite responses to food intake in lean and overweight dogs. Dogs were divided into two body condition groups: lean (BCS 4–5, *n* = 12) and overweight (BCS 6–8, *n* = 16), and the mixed model repeated measures analyses were applied. Values are given as liquid chromatography-time of flight mass spectrometry (LC-TOFMS) extracted ion chromatogram signal areas (mean ± SEM). Fasting plasma samples were taken 15 min before serving a test meal at time 0 (arrow) and metabolite signal areas in lean and overweight dogs are shown as response curves from fasting to 4 h after feeding. The overall carnitine (**a**) and acetylcarnitine (**b**) signal areas were lower in overweight compared with lean dogs. **b** At fasting, overweight dogs had lower plasma acetylcarnitine signal area than lean dogs. Lean dogs showed decreased acetylcarnitine signal area in response to food intake at one hour postprandially. Significant differences between time points (^¤¤¤^*P* < 0.001) and between body condition groups overall (**P < 0.01) are indicated. Different letters (a and b) indicate significant differences between body condition groups within time point (*P* < 0.05). In all dogs, propionylcarnitine (**c**) and stearoylcarnitine (**d**) increased in overall response to food intake, while vaccenylcarnitine (**e**) decreased (*P* ≤ 0.005 for all)

Based on previous findings of variations in lipid metabolism in overweight dogs [14, 35], a list of potentially interesting metabolites related to lipid metabolism (i.e. acylcarnitines and taurine) was created (*n* = 61) and these were searched for in plasma, based on compound accurate masses using the LC-TOFMS spectra generated. Six of the target metabolites were found to be above the limit of quantification (signal-to-noise ratio; S/*N* > 10) and were used in the statistical analyses (Fig. 1 and Additional file 5). Plasma responses of these six metabolites in the feed-challenge test were evaluated by mixed model repeated measures analysis in SAS [41–43] with significance level *P* < 0.05. In the statistical model, body condition group (lean and overweight) was defined as an independent variable and no baseline correction was conducted (i.e. the fasting value was included as a time point). The model analysed the response over time from fasting to four hours after feeding, and differences between the lean and overweight groups. Thus, the model is capable of overall and pair-wise comparisons, and corrects for multiple comparisons within the model by Tukey-Kramer adjustment. Logarithmic transformation of raw data was performed to correct for non-normality in the univariate statistical analysis of propionylcarnitine and stearoylcarnitine signal areas based on the appearance of the residuals within the mixed model. After Bonferroni correction for multiple comparisons, the level of significance was α < 0.008 (0.05/*n* = 6). To investigate the possibility of metabolic inflexibility in slightly overweight dogs (BCS 6) a mixed model repeated measurements model was constructed to analyse the acetylcarnitine response including three body condition groups (lean, slightly overweight and prominently overweight dogs) (Additional file 2).

#### Phospholipids

The presence of 317 phospholipids was determined in plasma by LC-TOFMS analysis and the amounts of these phospholipids were determined using four phospholipid internal standards. Phospholipids that had a value of zero in more than 50% of observations were excluded. To handle the remaining zero values, all observations in the dataset for the remaining 118 phospholipids (Additional file 6) had 0.01 nM added to the measured concentration. Multivariate statistics (SIMCA-P + 13.0 Umetrics, Umeå, Sweden) were used to identify phospholipids (nM) that varied with time in the feed-challenge test or were related to overweight (*n* = 118 as x-variables). Randomisation of raw data and step-wise removal of up to three outliers (using principal component analysis) in each comparison was applied.

A paired multivariate model, orthogonal partial least-squares effect projection analysis (OPLS-EP), was used to compare each postprandial time point with fasting. This model compares responses (here with the fasting concentration subtracted from each postprandial time point) as x-variables to a target value of y = 1 [40]. The OPLS-EP model expresses the structure of the data and can identify individuals with a deviating metabolic response to an intervention, here the feed-challenge test. Four OPLS-EP models were constructed using unit variance scaling (UVN) for x-variables and no scaling for the y-variable, as recommended by the model creators [40]. The significance of OPLS-EP models was verified using cross-validated analysis of variance (CV-ANOVA) [44], where a value of *P* < 0.05 was considered significant. To determine the most important discriminative phospholipids in significant OPLS-EP models, variable importance of projection (VIP) was used. Phospholipids with VIP *>* 1.5 and for which the corresponding jackknife-based 95% confidence intervals (CI) were not close to or including zero were considered discriminative and significant for the observed separations. The phospholipids with significant VIPs were tested with mixed model repeated measures analysis as described above. After Bonferroni correction for multiple comparisons, the level of significance was α < 0.004 (0.05/*n* = 12).

Another multivariate model, orthogonal partial least-squares discriminant analysis (OPLS-DA), was used to detect any differences between the pre-defined classes (lean and overweight dogs) in five different models (fasting, 1, 2, 3 and 4-h postprandially) using pareto scaling. The OPLS-DA model expressed the covariation between the phospholipid data and lean and overweight group of dogs, as well as the orthogonal variation that was not related to classes.

According to the hypothesis and based on previous findings of fasting metabolic variations in overweight humans [45], four phosphatidylcholines (PCaa; C38:4, C38:5, C40:5 and C40:6) and two lysophosphatidylcholines (LPCa; C18:1 and C18:2) were selected from the fasting dataset and tested in linear regression analysis with BCS as x-variable. After Bonferroni correction for multiple comparisons, the level of significance was α < 0.008 (0.05/*n* = 6). Any phospholipid that was found to be significant in linear regression analysis was further tested for significant differences between body condition groups.

## Results

### Metabolites

The LC-TOFMS analyses identified six metabolites that were above the limit of quantification; carnitine, acetylcarnitine, propionylcarnitine, stearoylcarnitine, vaccenylcarnitine and taurine. In all dogs, mixed model repeated measures analyses showed that propionylcarnitine and stearoylcarnitine increased in overall response to food intake, whereas vaccenylcarnitine decreased (*P* ≤ 0.005 for all) (Fig. 1). None of these three metabolites differed between body condition groups. The overall carnitine signal area was lower in overweight compared with lean dogs, as was the acetylcarnitine signal area (both *P* ≤ 0.004) (Fig. 1). The carnitine signal areas did not change during the feed challenge in any of the groups. At fasting, the acetylcarnitine signal area was lower in overweight dogs than in lean dogs (*P* = 0.001). In addition, in lean dogs the acetylcarnitine signal area decreased from fasting to one hour postprandially (*P* < 0.0001), while in overweight dogs the signal area was not affected by food intake (Fig. 1). The overall taurine response did not change after food intake and the overall taurine response did not differ between lean and overweight dogs after correction for multiple comparisons (Fig. 1).

### Phospholipids

**Fig. 2:**
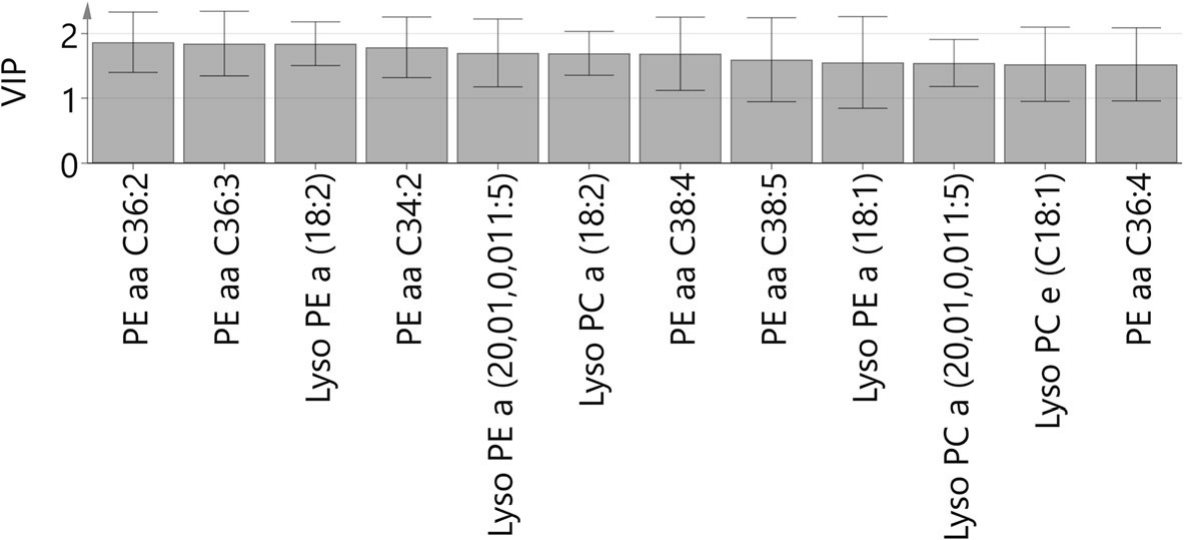
Phospholipid responses to food intake. Variable importance of projection (VIP) values, based on orthogonal partial least-squares effect projection (OPLS-EP) analysis (4-h time point minus fasting) of the phospholipid dataset. Values are displayed as VIP and confidence interval (CI). Discriminant phospholipids adding significant structure to the model (VIP > 1.5) and for which the corresponding jackknife-based 95% CIs were not close to or including zero are displayed

**Fig. 3:**
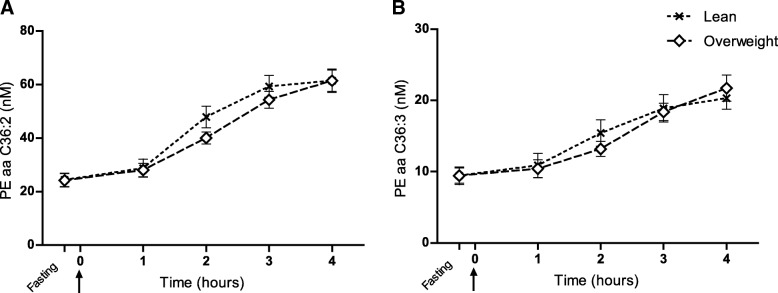
Phospholipid responses to food intake in lean and overweight dogs. (**a** and **b**) Fasting plasma samples were taken 15 min before serving a test meal at time 0 (arrow) and phospholipid concentrations in lean (BCS 4–5, *n* = 12) and overweight (BCS 6–8, *n* = 16) dogs are shown as response curves from fasting to 4 h after feeding. Mixed model repeated measures analyses were applied and showed increasing responses to food intake (overall significant for both between time points, *P* < 0.0001), but no differences between body condition groups. Values are given as nM concentrations (mean ± SEM). PEaa C36:2; phosphatidylethanolamine with 36 carbons, two ester bonds (aa) and two double bonds. PCaa C36:3; phosphatidylethanolamine with 36 carbons, two ester bonds and three double bonds. Note: A and B were the most discriminant phospholipids over time between fasting and the 4-h postprandial time point according to variable importance of projection analysis (VIP) in Fig. 2

The LC-TOFMS analysis quantified 118 phospholipids that were present in more than 50% of the plasma samples and these were used in multivariate models for comparisons between time points and between lean and overweight groups of dogs. In the paired multivariate OPLS-EP analyses, three models (i.e. fasting concentration subtracted from the 2, 3 and 4-h postprandial time points) were significant (*P* < 2.8 × 10^− 5^ for all). Visual interpretation of the significant OPLS-EP models did not reveal any cluster of dogs with a deviating phospholipid response to the feed challenge, and lean and overweight groups could not be visually separated. The most predictive OPLS-EP model (one predictive component and two orthogonal components; Q^2^ 0.91, R^2^Y 0.96, CV-ANOVA *P* = 4.9 × 10^− 9^) was the fasting concentration subtracted from the 4-h postprandial time point. Based on this OPLS-EP model, 12 significant VIPs were identified (Fig. 2). Mixed model repeated measures analysis of the 12 discriminant phospholipids showed that nine of them had overall increasing responses after food intake (PEaa C36:2, PEaa C36:3, LysoPEa 18:2, PEaa C34:2, LysoPEa 20.01.0.001:5, LysoPCa 18:2, PEaa C38:4, PEaa C38:5 and PEaa C36:4) (*P* ≤ 0.003 for all). The level of significance was α < 0.004 (0.05/*n* = 12) after Bonferroni correction for multiple comparison. Although significant overall over time, none of these nine phospholipids differed between fasting and the 1-h postprandial time point or differed in response between body condition groups. The two most significant phospholipids over time according to VIP values are shown in Fig. 3.

**Fig. 4:**
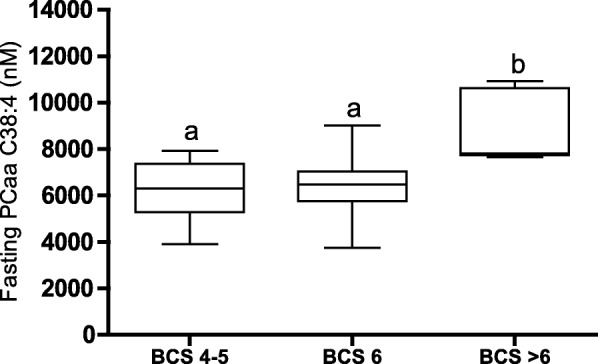
Differences in fasting phosphatidylcholine (PCaa C38:4) concentrations between body condition groups. Dogs were given a clinical body condition score (BCS 4–8 on a 9-point scale, *n* = 28), and were then divided into three body condition groups based on linear regression analysis (see Results section). One-way ANOVA (*P* = 0.003) was performed and differences in fasting PCaa C38:4 (nM) concentrations between lean (BCS 4–5, *n* = 12), slightly overweight (BCS 6, *n* = 10) and prominently overweight dogs (BCS > 6, *n* = 6) are shown as a box plot (median fasting values, 25–75% percentile, whiskers min. to max.). Different letters (a and b) indicate body condition groups that differed significantly (*P* < 0.05)

The multivariate OPLS-DA models at each separate time point did not identify any multivariate difference in phospholipid profiles between lean and overweight dogs. The hypothesis-driven linear regression analyses showed a positive association between one fasting phosphatidylcholine (PCaa C38:4) and BCS (R^2^ 0.31 and *P* = 0.002). Although the linear regression analysis was significant, the plot indicated no differences in PCaa C38:4 concentrations between lean (BCS 4–5) and slightly overweight dogs (BCS 6). To investigate this further, the dogs were divided into groups of lean (BCS 4–5, n = 12), slightly overweight (BCS 6, *n* = 10) and prominently overweight dogs (BCS > 6, *n* = 6) for this comparison and a one-way ANOVA with Tukey-Kramer adjustment was used. Prominently overweight dogs (BCS > 6) had significantly higher PCaa C38:4 concentration (*P* < 0.05) compared with both lean (BCS 4–5) and slightly overweight dogs (BCS 6) (Fig. 4). Age, body weight, calculated ideal body weight in overweight dogs and test-meal size for the tree body condition groups (lean, slightly overweight and prominently overweight dogs) are shown in Additional file 2.

## Discussion

In this study, an acetylcarnitine pattern indicating metabolic inflexibility to food intake was identified in spontaneously overweight Labrador Retriever dogs although none of the overweight dogs displayed signs of profound insulin resistance [39]. Overall carnitine signal areas were found to be lower in overweight dogs than in lean dogs. Interestingly, a lower carnitine status could theoretically slow down fatty acid oxidation as carnitine transports fatty acids inside of the mitochondria for beta oxidation [46].

Overweight dogs showed lower acetylcarnitine signal area than lean dogs at fasting. In addition, acetylcarnitine decreased at one hour postprandially in lean dogs, while in overweight dogs there was no response to food intake. Acetylcarnitine concentration as a measure of a flexible metabolism has been suggested [16, 47, 48], as acetyl-CoA is mainly derived from fatty acid oxidation but may also be generated from glucose or amino acid catabolism [49]. Consequently, acetylcarnitine can be formed from all substrates used for mitochondrial oxidation. The term metabolic inflexibility has been introduced to describe a decreased capacity of the mitochondria to switch freely between carbon fuels derived from different substrates [19] and metabolic inflexibility has been proposed as a link between obesity and insulin resistance in other species [17, 49, 50]. However, even though overweight dogs often become insulin-resistant, to our knowledge acetylcarnitine concentrations in dogs have not been investigated previously in a feed-challenge test. The lower carnitine and acetylcarnitine signal areas in overweight compared with lean dogs might be inter-related, as carnitine, acetyl-CoA and the enzyme acetyltransferase are all needed to produce the most abundant acylcarnitine, namely acetylcarnitine [16]. Consequently, the overall lower acetylcarnitine signal area in overweight dogs (but not the lack of postprandial decrease) might be explained by low availability of free carnitine that could subsequently lead to a lower fatty acid oxidation rate. However, with the present non-longitudinal study design, it was not possible to determine whether a potential carnitine insufficiency was a cause or a consequence of the spontaneous overweight.

In the fasting state, the most commonly used substrate for oxidation is fatty acids, oxidised to meet the energy demand during feed deprivation [50]. However, it has been proposed that overweight subjects might oxidise more than one substrate at a time due to mitochondrion overload and thereby decrease the total mitochondrial oxidation rate, thus showing lower acetylcarnitine concentrations compared with lean subjects [18]. It has also been suggested that obesity might cause increased but incomplete beta oxidation where obese individuals show higher acetylcarnitine and higher long-chain acylcarnitine concentrations than lean [50]. The lower fasting acetylcarnitine signal area in overweight compared with lean dogs in the present study thus indicate decreased mitochondrial oxidation rate of all substrates or decreased fatty acid oxidation in particular. This indication of a decreased oxidation pattern contradicts the pattern of increased but incomplete fatty acid oxidation that has been demonstrated by increased acylcarnitine concentrations in overweight humans [25] and acutely obese high-fat fed rodents and dogs [14, 50]. The fact that the overweight dogs of the present cohort were rather chronically than acutely overweight and no acute high-fat feeding had been conducted, might partly explain the different acylcarnitine and oxidation pattern between studies.

In addition, lean dogs of the present study showed significantly decreased acetylcarnitine signal area from fasting to one hour after food intake, while overweight dogs showed no response to feeding. These findings indicate a flexible metabolism in lean dogs that was not seen in overweight dogs. The decreased postprandial acetylcarnitine signal area in lean dogs could represent the fuel switch from using endogenously stored lipids to using recently absorbed glucose from the test meal, as has previously been shown in humans [26]. Thus, the transition from a catabolic to an anabolic state was evident in lean, but not in overweight dogs. Lean (BCS 4–5) and slightly overweight dogs (BCS 6) displayed comparable leptin concentrations (Additional file 2). However, signs of metabolic inflexibility was present already in slightly overweight dogs (BCS 6), as shown by their acetylcarnitine response (Additional file 2). Interestingly, the acetylcarnitine response did not differ between slightly overweight (BCS 6) and prominently overweight dogs (BCS > 6) indicating a comparable metabolic inflexibility to food intake in these two groups. These findings suggest that metabolic inflexibility in dogs might be influenced by other factors than just adiposity (Additional file 7).

Metabolic inflexibility has been considered a link between overweight and insulin resistance in humans and might be included as part of the human metabolic syndrome, which predisposes for development of cardiovascular disease and type 2 diabetes mellitus [51–53]. Whether metabolic inflexibility should be considered a potential health risk in dogs, as in humans, is currently not known. Metabolic inflexibility might become more severe with decreased physical activity as shown in bed-rested humans [51] and by chronic overfeeding which may cause mitochondrion overloads as shown in rodent models [18]. The occurrence of a metabolic syndrome in dogs is debated [54] but metabolic features in overweight and obese dogs also recognised in the human metabolic syndrome have been described in a few dog studies [11, 12, 55]. The major difference between species probably lies in the co-morbidities. There is currently little evidence that overweight dogs develop cardiovascular diseases other than hypertension [56], or type 2 diabetes mellitus [10, 57] as consequences of meeting the canine metabolic syndrome inclusion criteria. A hypothesis figure showing possible relationships between suggested factors in the development of insulin resistance and/or metabolic syndrome in humans, rodents and dogs has been constructed (Additional file 7). As metabolic inflexibility was found already in slightly overweight dogs in this study, overweight could be a possible confounder, a mediator or both in the development of metabolic inflexibility in dogs. Once developed, metabolic inflexibility has been suggested as a possible driver for overweight in the hypothesis figure. Interestingly, none of the overweight dogs showed signs of profound insulin resistance [39] although their acetylcarnitine response indicated metabolic inflexibility.

It has been suggested that carnitine requirements increase under conditions of sustained metabolic stress, such as in obese rodents [18, 58]. The exact reason for this is not known but it is possible that constant over-nutrition could lead to overloaded mitochondria, lower oxidation rates and higher metabolic stress in the overweight dogs in this cohort. In fact, the prominently overweight dogs (BCS > 6) in the present cohort showed higher urinary cortisol/creatinine ratio in morning urine than lean dogs [39] which could be associated with metabolic stress. In a long-term study of rats, it was found that high-fat feed caused decreased carnitine concentrations with simultaneous diminished expression of carnitine biosynthetic genes, disturbances which were reversed with carnitine supplementation [47]. All dogs ate complete commercial diets that had animal proteins as main protein source making protein related carnitine insufficiency unlikely. A limited number of lean and overweight dogs ate complete diets where the main energy source came from fat and the frequency with which lean and overweight dogs were given other foods than complete diets did not differ between groups (Additional file 1). Carnitine depletion in overweight subjects due to excessive long-chain acylcarnitine production has also been suggested in rodents [47] and increased long-chain acylcarnitines have been found in acutely overweight dogs [14]. The latter pattern was not observed in overweight dogs in the present study, where two long-chain acylcarnitines were detected but with equally low signals in both lean and overweight dogs. A possible reason for these diverging results between studies on dogs might be that our chromatographic conditions were not completely optimised towards detection of long-chain acylcarnitines, as these compounds co-elute with phospholipids [59]. Furthermore, our dogs were chronically rather than acutely overweight, which might affect both carnitine metabolism and fatty acid oxidation. We suggest that the lower carnitine status in the overweight dogs might be related to adiposity and possible metabolic stress although influence of dietary differences cannot be completely ruled out due to the study design. To evaluate the origin of potential carnitine insufficiency in overweight dogs and its effect on lipid metabolism, feeding trials using both acute and chronic models of canine overweight are needed.

While carnitine and acetylcarnitine differed between body condition groups, the more extensive phospholipid dataset showed no multivariate separation between lean and overweight dogs. Similarly, in a previous human study, half the variation in a multivariate model of body mass index was explained by acylcarnitines and to a much lesser extent by phosphatidylcholines [60]. Phosphatidylcholines have been shown to be the most abundant phospholipids in dogs [32]. Therefore, it was not surprising that the phosphatidylcholines comprised the main variation related to body condition score within the phospholipid dataset in the present study, although without separating body condition groups. Results from a previous study on the same cohort of dogs indicated impaired exogenous handling of dietary triglycerides with increased concentrations postprandially in the overweight group of dogs [39]. In the present study, overweight and lean dogs did not differ in phospholipid profiles, which primarily represent the endogenous lipid pathway. Lipoprotein fractions were not measured in the present study, but previous studies in dogs show diverging results regarding lipoprotein changes in overweight [28–31]. Lipoprotein changes in overweight dogs need further evaluations as such changes could influence the composition of the plasma phospholipid profile although no multivariate separation between body condition groups was found in the phospholipid dataset of the present cohort. Studies on humans have found positive associations between one particular phosphatidylcholine (PCaa C38:4), body mass index and waist circumference [61], associations that persisted even when the effect of lipoprotein fractions was accounted for [62]. In the present study, the hypothesis-driven linear regression analysis showed a positive association between fasting PCaa C38:4 and BCS. Comparing groups, prominently overweight (BCS > 6), but not slightly overweight dogs (BCS 6), differed from lean dogs (BCS 4–5). In overweight humans, elevated phosphatidylcholines (e.g. PCaa C38:4) have been shown to be associated with insulin resistance, and lipotoxicity [45] or pro-inflammatory properties of the compounds [34] have been proposed as the underlying cause. Overweight dogs of the present cohort did not differ from lean dogs in terms of postprandial insulin response, insulin sensitivity assessed by a fasting homeostasis model of assessment [39] or, in inflammatory status measured by high-sensitivity C-reactive protein [63], which could possibly be explained by the rather moderate overweight in the present cohort. Further studies including obese dogs and using more sensitive or dynamic measures of insulin sensitivity are warranted to elucidate the possible role of elevated PCaa C38:4 and metabolic inflexibility in the metabolism of overweight dogs.

In the present study, nine phospholipids, mostly phosphatidylethanolamines, were shown to be related to food intake in mixed model repeated measures analyses. In a previous oral lipid challenge in humans, phosphatidylethanolamines (PEaa C36:2 and PCaa C36:3) showed an almost two-fold increase at two hours postprandially [64]. Interestingly, the present dog cohort displayed a comparable increase in the same phospholipids in response to the high-fat feed-challenge test. These phosphatidylethanolamines could thereby be related to exogenous lipid metabolism and recent fat intake [64]. Concerning acylcarnitines, the postprandial response has been postulated to depend on chain length and saturation. The results largely followed earlier suggested patterns, as the short-chain propionylcarnitine and the long-chain stearoylcarnitine increased in response to food intake, while acetylcarnitine decreased and free carnitine was unaffected, which resembles observations made in humans after a meal challenge [26, 65]. The increase in stearoylcarnitine could be related to recent fat intake and oxidation, while the elevation in propionylcarnitine could presumably derive from amino acids present in the test meal [66]. The unsaturated long-chain vaccenylcarnitine instead showed a decrease in response to food intake, which could be explained by proposed higher oxidation of unsaturated compared with saturated ester compounds after food intake [65, 67].

Despite great efforts, a relatively low number of dogs could finally be included. However, the paired multivariate models and the mixed model repeated measurements analyses used in the present study proved to be robust and were able to detect clear responses over time and group differences in the feed-challenge test. It would have been desirable to include more obese (BCS 8–9) but yet healthy dogs, but such dogs proved extremely difficult to enrol. An objective confirmation (e.g. dual energy X-ray absorptiometry) of the clinically assessed BCS might have strengthened the BCS results, but this method was not available at the time of the study. The fact that dogs from only one breed and sex were used in this study has presumably reduced the individual variation and made it possible to detect subtle metabolic variations despite a relatively small sample size. However, to further investigate the concept of metabolic inflexibility in canine overweight, studies on larger cohorts of various breeds and of a wider variety of body condition scores are needed. Furthermore, possible effects of different background feeding regimens need to be investigated and substrate oxidation studies are warranted.

## Conclusions

This study on spontaneously overweight healthy Labrador Retrievers showed that even a moderate overweight in dogs can influence lipid metabolism parameters. Overweight dogs showed overall compromised carnitine status representing a potential carnitine insufficiency. The acetylcarnitine response in overweight dogs indicated decreased fatty acid oxidation at fasting and metabolic inflexibility to food intake. Further studies on metabolic inflexibility and its potential role in the metabolism of overweight dogs are warranted.

## Additional files

Additional file 1: Background diet in home environment of the 28 Labrador Retriever dogs included in the study. (PDF 357 kb)

Additional file 2: Acetylcarnitine and leptin concentrations related to body condition scores. (PDF 560 kb)

Additional file 3: Test-feed analysis, as fed. (PDF 302 kb)

Additional file 4: Composition of the phospholipid standard mixture used for extraction of the dog plasma samples. (PDF 260 kb)

Additional file 5: Metabolite dataset (signal areas), *n* = 6. (XLSX 17 kb)

Additional file 6: Phospholipid dataset (nM), *n* = 118. (XLSX 238 kb)

Additional file 7: Hypothesis figure. (PDF 358 kb)

## Abbreviations

BCS: Body condition score
CI: Confidence interval
CV-ANOVA: Cross-validated analysis of variance
DER: Daily energy requirements
LC-TOFMS: Liquid chromatography-time of flight mass spectrometry
LPC: Lysophosphatidylcholine
LPE: Lysophosphatidylethanolamine
OPLS-DA: Orthogonal partial least-squares discriminant analysis
OPLS-E: Orthogonal partial least-squares effect projection analysis
PC: Phosphatidylcholine
PE: Phosphatidylethanolamine
SD: Standard deviation
VIP: Variable importance of projection
