# Supplementary material for: Indication of metabolic inflexibility to food intake in spontaneously overweight Labrador Retriever dogs
Source: BMC Vet Res. 2019 Mar 20;15:96. doi: 10.1186/s12917-019-1845-5 (PMC6425671; doi:10.1186/s12917-019-1845-5)
Supplement: Supplementary file 2 — Acetylcarnitine and leptin concentrations related to body condition scores. (PDF 560 kb) [file 12917_2019_1845_MOESM2_ESM.pdf]

Additional file 2. Acetylcarnitine and leptin concentrations related to body condition scores.

To investigate possible metabolic variations in slightly overweight dogs (BCS 6) the cohort was divided into three body condition groups. Descriptive statistics for these groups are presented below in a table. A linear regression analysis was conducted to explore the association between the assigned clinical body condition score and fasting leptin concentrations in serum (see leptin graph below). The acetylcarnitine responses of the three body condition groups were analysed using mixed model repeated measurements (see acetylcarnitine graph below).

Descriptive statistics for the 28 Labrador Retriever dogs and the amount of test food given in the feed-challenge test*

	**Lean** (BCS 4-5) n=12	**Slightly overweight** (BCS 6) n=10	**Prominently overweight** (BCS >6) n=6
Age *(year)*	5.3 ± 1.4[a]	4.6 ± 1.4[a]	6.2 ± 1.6[a]
Body weight *(kg)*	34.8 ± 2.5[a]	36.9 ± 2.3[ab]	43.9 ± 4.2[b]
Ideal body weight***(kg)*	34.8 ± 2.5[a]	34.4 ± 2.2[a]	39.2 ± 2.7[b]
Test-meal size*** *(g)*	222 ± 12[a]	220 ± 11[a]	243 ± 12[b]

*Variables are expressed as mean ± SD. Within each row, values with different superscript letter (a or b) differ significantly (*P*<0.05).

**Ideal body weight of overweight dogs was calculated as previously described (Verkest *et al.*, 2011; Laflamme, 1997).

***Test diet: Hills Science Plan^{TM} Canine Adult Performance.

Leptin graph

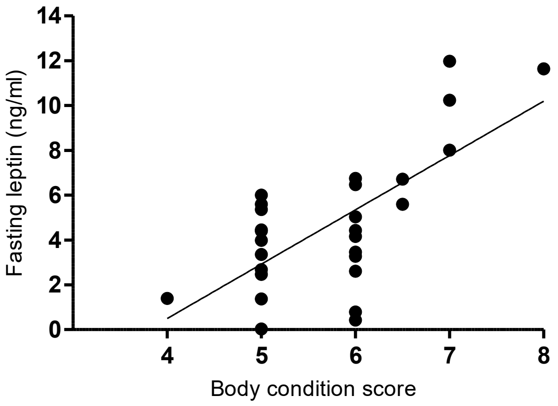

Dogs were assigned a clinical body condition score (BCS). Leptin concentration measured by Canine Leptin ELISA, Millipore, MO, in fasting serum samples as a function of the assigned body condition score (significant association, linear regression R^2=0.41, *P*<0.0001). Note the comparable leptin concentrations between lean dogs (BCS 4-5) and slightly overweight dogs (BCS 6).

Acetylcarnitine graph

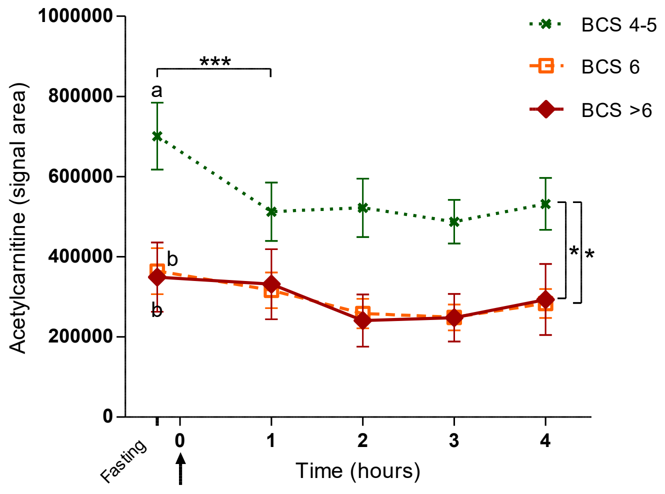

Dogs were divided into three body condition scoring (BCS) groups: lean (BCS 4-5, n=12), slightly overweight (BCS 6, n=10) and prominently overweight (BCS>6, n=6) and a mixed model repeated measures analysis was applied. Values are given as liquid chromatography-time of flight mass spectrometry (LC-TOFMS) extracted ion chromatogram signal areas (mean ± SEM). Fasting plasma samples were taken 15 minutes before serving a test meal at time 0 (arrow) and metabolite signal areas in lean, slightly overweight and prominently overweight dogs are shown as response curves from fasting to 4 hours after feeding. Significant differences in overall responses between and within body condition groups are indicated by asterisks (**P*<0.05, ****P*<0.001). Different letters (a and b) indicate significant differences between body condition groups within time point (*P*<0.01).
