# Supplementary material for: Indication of metabolic inflexibility to food intake in spontaneously overweight Labrador Retriever dogs
Source: BMC Vet Res. 2019 Mar 20;15:96. doi: 10.1186/s12917-019-1845-5 (PMC6425671; doi:10.1186/s12917-019-1845-5)
Supplement: Supplementary file 3 — Test-feed analysis, as fed. (PDF 302 kb) [file 12917_2019_1845_MOESM3_ESM.pdf]

Additional file 3. Test-feed analysis, as fed [a]

Analysis	**Results** (deviation)	Method/Reference	Lab
LW03P Ash	5.89 g/100 g (± 10%)	2009/152/EU modified	EUSELI
LP022 Crude protein	28.2 g/100 g (± 7%)	Dumas (Nx6.25)	EUSELI
LP00W Alanine	17.3 g/kg (± 8%)	SS-EN ISO 13903:2005	EUSELI
LP00W Arginine	19.4 g/kg (± 8%)	SS-EN ISO 13903:2005	EUSELI
LP00WAsparagine and Asparagine acid	22.3 g/kg (± 8%)	SS-EN ISO 13903:2005	EUSELI
LP00W Cystine	7.3 g/kg (± 8%)	SS-EN ISO 13903:2005	EUSELI
LP00W Fenylalanine	12.6 g/kg (± 8%)	SS-EN ISO 13903:2005	EUSELI
LP00W Glutamine acid	38.1 g/kg (± 8%)	SS-EN ISO 13903:2005	EUSELI
LP00W Glycine	24.1 g/kg (± 8%)	SS-EN ISO 13903:2005	EUSELI
LP00W Histidine	5.1 g/kg (± 8 %)	SS-EN ISO 13903:2005	EUSELI
LP00W Hydroxiproline	6.0 g/kg (± 8%)	SS-EN ISO 13903:2005	EUSELI
LP00W Isoleucine	13.1 g/kg (± 8%)	SS-EN ISO 13903:2005	EUSELI
LP00W Lysine	13.6 g/kg (± 8%)	SS-EN ISO 13903:2005	EUSELI
LP00W Metionine	4.4 g/kg (± 8%)	SS-EN ISO 13903:2005	EUSELI
LP00W Ornitine	0.3 g/kg (± 8%)	SS-EN ISO 13903:2005	EUSELI
LP00W Proline	22.1 g/kg (± 8%)	SS-EN ISO 13903:2005	EUSELI
LP00W Serine	19.3 g/kg (± 8%)	SS-EN ISO 13903:2005	EUSELI
LP00W Threonine	11.9 g/kg (± 8%)	SS-EN ISO 13903:2005	EUSELI
LP00W Tyrosine	9.0 g/kg (± 8%)	SS-EN ISO 13903:2005	EUSELI
LP00W Valine	17.1 g/kg (± 8%)	SS-EN ISO 13903:2005	EUSELI
LP00W Sum amino acids	286.7 g/kg	SS-EN ISO 13903:2005	EUSELI
LP089 Crude fat	23.2 g/100 g (± 10%)	EU DIR 98/64 modified	EUSELI
LP05C Total dietary fibre	8.83 g/100 g (± 15%)	AOAC 985.29	EUSELI
LP05M Total sugar	7.0 g/100 g (± 15%)	Total sugar by Ekelund [b]	EUSELI
MJ010 Starch	24.30 %	In house method [c]	EUNOTR2
LW01M Dry matter	92.7 g/100 g (± 10%)	2009/152/EU modified	EUSELI

[a] Analysis performed by Eurofins Food & Agro Testing Sweden AB, Lidköping, Sweden. www.eurofins.se
[b] Ekelund Sigvard, Statens lantbrukskemiska kontrollanstalt Meddelande 28 (1966), bilaga VIII
[c] Simple sugars not included

Ash (A) from analysis: 5.6 g/100g
Crude protein (CP) from analysis: 28.2 g/100g
Crude fat (CF) from analysis: 23.2 g/100g
Dietary fibre (DF) from analysis: 8.8 g/100g
Nitrogen free extract (NFE) from analysis: Total sugar 7.0 g/100g + Starch 24.3 g/100g = 31.3 g/100g

Calculated NFE from test-feed analysis
NFE: 100 - (A 5.6 + CP 28.2 + CF 23.2 + DF 8.8) = 34.2 g/100 g (as fed)

Calculated metabolisable energy (ME) according to test-feed analysis: 420 kcal/100g (as fed)
(Atwater factors used: CF 8.7, CP 3.5 and NEF 3.5)
28.2*3.5 = 98.7/420 (23.5% of ME as protein)
23.2*8.7 = 201.84/420 (48% of ME as fat)
34.2*3.5 = 119.7/420 (28.5% of ME as calculated NFE from analysis)

31.3*3.5 = 109.6/415 (27% of ME as NFE from analysis) (gives 410 kcal/100g as fed)

ME according to manufacturer: 423 kcal/100g (as fed)
23% of ME as protein
51% of ME as fat
26% of ME as NFE
