# Supplementary material for: Indication of metabolic inflexibility to food intake in spontaneously overweight Labrador Retriever dogs
Source: BMC Vet Res. 2019 Mar 20;15:96. doi: 10.1186/s12917-019-1845-5 (PMC6425671; doi:10.1186/s12917-019-1845-5)
Supplement: Supplementary file 4 — Composition of the phospholipid standard mixture used for extraction of the dog plasma samples. (PDF 260 kb) [file 12917_2019_1845_MOESM4_ESM.pdf]

Additional file 4. Composition of the phospholipid standard mixture used for extraction of the dog plasma samples[1]

Lipid standard[2]	Monoisotopic molecular weight (g/mol)	Concentration (nmol/L)
LPC C13:0	453.2855	224.7
SM C12:0	646.5050	246.0
PE 17:0-14:1 (C31:1)	675.4839	71.30
PC 12:0-13:0 (C25:0)	635.4526	151.6

[1]The mixture was prepared in LC-MS grade methanol (LC-MS grade, VWR Chemicals, Radnor, PA).

[2]LPC lysophosphatidylcholine, SM sphingomyelin, PE phosphatidylethanolamine, PC phosphatidylcholine. phospholipid standards were obtained from (Avanti Polar Lipids, Alabaster, AL)
