# Supplementary material for: Indication of metabolic inflexibility to food intake in spontaneously overweight Labrador Retriever dogs
Source: BMC Vet Res. 2019 Mar 20;15:96. doi: 10.1186/s12917-019-1845-5 (PMC6425671; doi:10.1186/s12917-019-1845-5)
Supplement: Supplementary file 7 — Hypothesis figure. (PDF 358 kb) [file 12917_2019_1845_MOESM7_ESM.pdf]

Additional file 7. Hypothesis figure

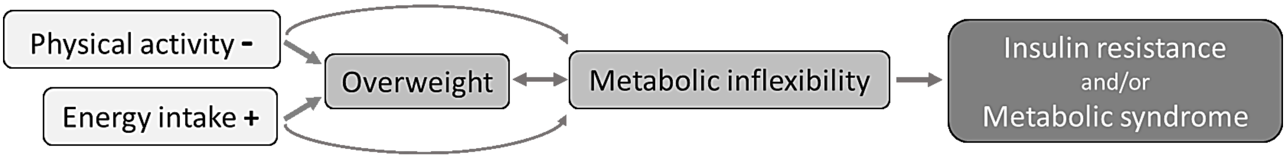

A hypothesis figure showing possible relationships between suggested factors in the development of insulin resistance and/or metabolic syndrome in humans, rodents and dogs. Drawing JS.
